# The Property of Alcohol Which Allures the Neurotic to Drink

**Published:** 1885-02-20

**Authors:** T. L. Wright

**Affiliations:** Bellefontaine, Ohio


### THE PROPERTY OF ALCOHOL WHICH ALLURES THE NEUROTIC TO DRINK.

By T. L. Wright, M.D., Bellefontaine, Ohio.

It has been the opinion of men for decades and centuries, and for aught I know, "from the beginning," that the primary and most obtrusive exhibition of the alcoholic influence affords an adequate solution of the motive which actuates the inebriate when he invokes intoxication. It has been supposed that the stimulation of the passions, the exaggeration of the ideas, the domination and enthronement of sensuality, represent the incitements which provoke to drunkenness. It has been the conspicuous sentiment of mankind, with but few exceptions, that these incentives to inebriation were the outcome of depravity of mind and lust of flesh, which were wholly within the dominion of the will and the capacity for restraint. The conclusion thence has been almost universal that the practice of alcoholic inebriety, in view of even its more manifest, and superficial, and circumscribed evils, is an audacious and detestable display of the most revolting characteristics with which the mind and body and soul of any human being can be imbued.

Against such sentiments Dr. Rush, and perhaps a few others, have, in the past, made some protest. Yet the general opinion as to the moral nature of the drunkard has remained, up to a very recent period, unchanged.

Latterly—within, perhaps, half a dozen years—there has been a questioning of the truth of the previous doctrines of mankind upon this subject. Depravity may be punished and restrained through fear; and sensuality may be rebuked, and its exhibition deterred in various ways. But it is found that punishment is of no practical avail in abolishing intemperance; and rebukes and shame are ineffectual in diminishing drunkenness.

It is a curious and an instructive sight which greets the eye as recent changes in the world's opinion of inebriety come under review. At first protests from careful minds were offered. The old notions as to the culpability of inebriety were cautiously examined. Several inquirers began to hint that alcohol was sought after and demanded—not for stimulation, not for exaggeration, not for ravening lust—but for repose, for quiet, for rest.

Here was a revolution indeed; a complete upsetting of all the antiquated ideas on the subject, and a substitution of new and rational principles. And this revolution, too, was fundamental, not superficial. It permeated all the questions and responsibilities allied to intemperance; and it placed the inebriate and his surroundings—the drunkard and the general public—in new relationships altogether. The writer of this, adopting the hints and teachings of such men as Drs. Parrish, Crothers and Hughes, has for several years advocated the doctrine that the inebriate is, as a rule, a constitutional neurotic; and that his inebriety, when turned towards alcohol, was so directed in search of an escape from a morbid excitement, not an entrance into one.

Under such views, the whole subject becomes clothed with new thoughts and new principles. The primal cause of intemperance is no longer sought exclusively within the organization of the drunkard himself. The unavoidable impressions resulting in a neurotic constitution, and in inebriety, pertain very often to the great and controlling agencies which pervade the universe. Sometimes, it is true, a neurotic predisposition is founded originally by wounds and physical accidents. But anything which has power to strongly impress and warp the constitution may produce a parallel result. Thus heat and cold, and the electrical conditions, may superinduce inebriety. The occult powers operating in the development of the various morbid states which affect the organization of mankind may work in a similar manner. Disturbances in the equilibrium of the magnetic medium, such as are not uncommon from agitations in the sun, may unquestionably prove profoundly effective in impressing the nervous constitution of man. In short, the primary causes of inebriety may quite frequently be referred to the operation of those great forces of nature, which, while they preside over the growth and health of the body, do, at the same time, determine its decay and modification.

From these general observations many conclusions are legitimately derived. The originating forces resulting in the neurotic temperament being oftentimes situated exterior to the human body, the responsibility for drunkenness, as well as the point of reformation, must also. not infrequently, be placed exterior to the human organization and beyond the operation of the human will. It is, therefore, an error held by many that a few years of enforced abstinence from alcoholic indulgence will eradicate intemperance from the earth; for wounds, accidents, diseases and the innumerable sources of the inebriate predisposition would remain in active force; and were it possible to remove every drunkard out of existence they would soon fill the world again with countless inebriates.

To refrain from alcoholic indulgence is, obviously, not always within the voluntary power of the neurotic inebriate. One or two illustrations of this fact, however, may not be out of place. Every man of mature years has seen instances similar to those following:

"Major W——, forty years old, a printer, had passed through the civil war with credit as a soldier. He was a man of more than average intelligence. Periodically he was a hard drinker, furious with drunkenness for days, and then sober and remorseful for weeks. He made every promise and every effort to abstain permanently from liquor. He was engaged to be married, under the condition that he would control his besetting propensity for a given time. Still he would drink and spree, always remorseful thereafter and pledging himself anew. At last, realizing the futility of his endeavors, despairing and ashamed—when sober and sorrowful—he walked out to a lonely spot in the darkness of night and, casting himself before an advancing train of freight cars, he met his fate."

Another instance:

"W. L—— was also a soldier during the late war. These poor inebriates are often soldiers who have borne the shocks and hardships, and diseases and wounds of war. Their nervous powers have been unsettled and thrown permanently out of equipoise. This man was a spasmodic drunkard. Becoming a government clerk in Washington, he was peculiarly subjected to temptation. He repeatedly tried to reform. He would sign the pledge, and solemnly promise himself and friends to quit drinking. At length, after an unavailing warfare against a constitutional predisposition, extending over a period of more than twenty years, at the end of a debauch, when all about him thought he would soon return to duty, he drew his razor across his throat, and so died."

In such cases as these, when the supreme moment comes, wherein self-respect and the opinion of the world demand decisive action, we see— not reformation, but suicide.

The discussion of the anæsthetic powers of alcohol has opened to view an entirely new field for inquiry. Indeed, in relation to the principles which are associated with the alcoholic temperament, the consideration of anæsthesia is like entering upon a new world. Eminent men are daily assuming the study and discussion of the anæsthesia of alcohol, as it presents itself in its manifold modifications. The recent paper by Prof. Palmer, in the Journal of Inebriety, upon alcohol as a paralyzing* agent is a good disquisition upon one of the many phases of the subject.

The relentless unrest of the constitutional neurotic; the ever-acting erethism of the weakened and poorly-nourished nervous system; the agonizing appeal for repose which goes forth from the neuræsthenic—ever thinking, and with no power to rest or change: these are some of the conditions hurrying the mind to insanity, which turn to anæsthesia and to alcohol for help. They seize upon strong drink as their best and only friend.

It is a great step toward unveiling the truth to recognize the fact that anæsthesia is, indeed, that property of alcohol which is paramount in importance; and it is a substantial advancement to discover that the soothing and lethal property of alcohol is that which so imperiously demands the homage and worship of the helpless neurotic. This vindicates, to some extent, the manliness even of disease, for it shows that it is not always the riot of lust, or the satisfaction of inordinate and maniacal stimulation, which incites the inebriate when he seeks intoxication.—*Alienist and Neurologist.*

*To be strictly accurate, the property of alcohol as a *paralyzant* was at first pointed out by Dr. T. W. Poole, of Ontario, in a book published by him in 1879. Dr. C. H. Hughes has also several times called attention to the same point.

